# Digital mobility assessment using wearable devices in people with Parkinson’s disease: a feasibility and implementation study in Ghana (TraPCAf) compared with a UK cohort

**DOI:** 10.3389/fnagi.2026.1899045

**Published:** 2026-10-02

**Authors:** Metin Bicer, Cameron Kirk, Mary Agoriwo, Momodou Cham, Albert Akpalu, Njideka Okubadejo, Javad Sarvestan, Jack Lumsdon, Yasin Okkaoglu, Katie Brittain, Ríona McArdle, Lisa Alcock, Alison J. Yarnall, Natasha Fothergill-Misbah, Richard Walker, Lynn Rochester, Silvia Del Din, TraPCAf

**Affiliations:** 1 Translational and Clinical Research Institute, Faculty of Medical Sciences, Newcastle University, Newcastle upon Tyne, United Kingdom; 2 Department of Physiotherapy and Rehabilitation Sciences, School of Allied Health Sciences, University of Health and Allied Sciences, Ho, Ghana; 3 Richard Novati Catholic Hospital, Sogakope, Ghana; 4 Department of Medicine, University of Ghana Medical School, Accra, Ghana; 5 College of Medicine, University of Lagos, Lagos, Nigeria; 6 Population Health Sciences Institute, Newcastle University, Newcastle upon Tyne, United Kingdom; 7 Biostatistics Research Group, Faculty of Medical Sciences, Newcastle University, Newcastle upon Tyne, United Kingdom; 8 National Institute for Health and Care Research (NIHR) Biomedical Research Centre (BRC): Newcastle, Newcastle upon Tyne, United Kingdom; 9 Newcastle upon Tyne Hospitals NHS Foundations Trust, Newcastle upon Tyne, United Kingdom; 10 Northumbria Healthcare NHS Foundation Trust, Newcastle upon Tyne, United Kingdom

**Keywords:** Africa, digital health technology, digital mobility outcomes, gait, real-world, feasibility, Parkinson’s disease, wearable sensor

## Abstract

**Introduction:**

Life expectancy is rising in Africa, increasing Parkinson’s disease (PD) prevalence. PD in Africa is often underdiagnosed/undertreated due to limited specialists, awareness and medication access. Gait impairments are among the earliest and most disabling PD symptoms, yet their presentation in African populations is poorly understood. This study aimed to (1) compare real-world mobility between PD and older controls in a population from Ghana (GHA-PD and GHA-CL), (2) examine associations between digital mobility outcomes (DMOs) and clinical/demographic factors between GHA-PD and a PD cohort from a high-income European country (UK-PD), and (3) assess the acceptability of the measurement method in GHA-PD and GHA-CL.

**Methods:**

PD (*n* = 50) and controls (*n* = 30) in Ghana wore a wearable device (Axivity AX6) for seven days. Raw data were used to derive 24 DMOs using the Mobilise-D pipeline. An age-gender-matched PD and control cohort (UK-CL), based in United Kingdom, from another study was included for comparison. Differences were also analysed by controlling confounding factors.

**Results:**

Sixteen of 24 DMOs differed significantly (*p* < 0.05) between GHA-PD and GHA-CL (steps per day: 2153 vs. 10001, daily walking time: 0.43 vs. 2.07 hours). Mobility impairment was more pronounced in GHA-PD than UK-PD, with differences remaining in 18 DMOs after adjustment for confounding factors. Feasibility and acceptability were high, although discomfort was commonly reported.

**Discussion:**

Reduced mobility and gait quality in GHA-PD highlight risks of functional decline and loss of independence. Further research should investigate lifestyle, cultural, environmental and healthcare factors that might contribute to these disparities.

## 1 Introduction

Life expectancy across Africa has risen significantly, reaching 63.8 years in 2023, with sub-Saharan Africa projected to account for half of the human global population by 2050 (World Health Organization, 2024). This aging trend is expected to increase the burden of neurological conditions such as Parkinson’s disease (PD). Although PD prevalence is often reported as similar to, or lower than, that in Europe (Williams et al., 2018), these figures are likely underestimates due to small sample sizes, hospital-based data and cultural perceptions that frame symptoms as normal aging (Akinyemi, 2012). As a result of low awareness of PD, underdiagnosis and delayed diagnosis are common (Fothergill-Misbah et al., 2021). Despite inadequate diagnosis, the number of people with PD (PwP) is expected to increase by 292% in western sub-Saharan Africa alone by 2050 (Su et al., 2025). However, the region faces a severe shortage of neurologists and other specialists trained in neurological disorders (Williams et al., 2018). PD-specific medications such as levodopa are rarely available and often unaffordable (Subramanian et al., 2025; Williams et al., 2018). Currently, clinical approaches to diagnosing, managing and treating PD in Africa are not adequately equipped to address the current and growing healthcare burden (Holst et al., 2020), with significant implications for the quality of life of PwP and their carers.

Gait impairments are among the most disruptive mobility impairments experienced by PwP (Delgado-Ortiz et al., 2023). These changes are common and appear very early, usually in the prodromal phase (Deane et al., 2014; Wilson et al., 2020). Their consequences are severe, limiting safe and independent physical activity. Failure to treat gait symptoms could significantly increase risk of falls and sustaining a serious injury, alongside loss of independence increasing burden upon health services and caregivers. Cultural, environmental, and societal factors mean that the impact of PD on gait and walking activity is likely to vary by country (Busch et al., 2015; Ryu et al., 2006), so gait impairments should not be generalized across countries. Although gait and mobility have been characterized across other non-African populations, there is a need to accurately describe these in Africa against a reference sample.

As a cost-effective and efficient solution, measures of walking speed, step count and a range of clinically relevant mobility characteristics are collectively referred to as digital mobility outcomes (DMOs) (Rochester et al., 2020), and can now be quantitatively and continuously measured in real-world settings from low-cost, non-invasive wearable devices. DMOs offer a promising approach to improving the diagnosis and clinical management of PD in Africa. In populations from high-income countries’, DMOs in PD have been shown to identify real-world impairments in physical activity, walking speed and step lengths in comparison to controls (Kirk et al., 2025), predict early disease (Rehman et al., 2019) and monitor PD progression (Kirk et al., 2023; Mancini et al., 2025; Sotirakis et al., 2023). Alongside improving diagnostic accuracy, DMOs offer an unobtrusive solution that monitors aspects of health that are of relevance to PwP such as maintaining the ability to walk safely and independently in daily life (Deane et al., 2014; Delgado-Ortiz et al., 2023; Port et al., 2021), as well as increasing accessibility to clinical assessments allowing PwP to be assessed remotely.

While DMOs have been widely applied in Western populations, their use in African populations has been limited. Greater research funding in Western countries has driven the development of DMO technologies and algorithms (Kirk et al., 2025; Micó-Amigo et al., 2023), requiring additional efforts to overcome logistical challenges of device provision, data uploads, and remote processing in African settings. While feasibility has been demonstrated for people living with frailty in rural Tanzania (Del Din et al., 2025b), no studies have examined the clinical utility of DMOs in African populations with PwP. Within West Africa, Ghana represents a setting in which access to specialist neurological care remains limited and structured rehabilitation pathways for PD are underdeveloped. Prior work examining rehabilitation access for PwP in Ghana has identified substantial barriers to timely diagnosis, specialist follow-up, and objective monitoring of mobility (Agoriwo et al., 2025, 2024), Ghana therefore provides a relevant setting in which to explore the feasibility and clinical utility of digital mobility outcomes as complementary measures to routine clinical care. We acknowledge, however, that Africa is a large and diverse continent, and that findings from this study should not be assumed to generalize to other African populations without further validation. Establishing known-groups validity (distinguishing PwP from controls) or construct validity (associations with PD clinical scales) would support DMOs as complementary measures alongside clinical assessments, enabling more accurate diagnosis distinct from typical aging and continuous monitoring of severity and progression. This could address limitations by reducing waiting times for diagnosis and specialist follow-up, increasing available time for clinicians and extending assessments to those in remote areas.

The aims of this study were to:

(1) describe real-world DMOs in PWP from Ghana compared to older adult controls from Ghana and a cohort of PwP from the UK,
(2) determine PD-specific differences in PwP from Ghana compared to the UK through associations of real-world DMOs with clinical and demographic characteristics,
(3) explore the feasibility, useability and acceptability of real-world monitoring of mobility using wearable sensors in the Ghanian cohorts of PD and controls.

## 2 Materials and methods

### 2.1 Participants and sampling

This study includes a cross-sectional analysis involving participants assessed at baseline as part of the Transforming Parkinson’s Care in Africa (TraPCAf) study (Walker et al., 2023). A total of 50 PwP (GHA-PD) were recruited and assessed between December 2023 to June 2024. An additional cohort of 30 control participants (GHA-CL) without PD were recruited and assessed from December 2024 to January 2025. Participants with PD were eligible if they were aged 18 years or older and had received a clinical diagnosis of PD according to the Movement Disorder Society (MDS) criteria (Postuma et al., 2015) and in Hoehn and Yahr stages I-IV. The control group comprised age- and gender-matched community-dwelling individuals from the same population as the patient group. PwP were recruited from one urban site and one rural site. Urban site participants were drawn from the neurology clinic of a tertiary referral hospital in Accra while rural participants were recruited from the PD clinic of a district hospital in Sogakope. These PwP had access to neurologist care and rehabilitation services like physiotherapy and nutrition care. Controls were community-dwelling individuals, identified through community outreaches and durbars (traditional public gatherings). The cohort thus reflects both urban and rural contexts. PwP were conveniently sampled from the selected study sites, whereas controls were purposefully sampled to ensure age- and gender-matching with the patient group. As a pilot study, the sample may not represent national demographics however, it provides valuable baseline data to inform future research. Ethical approval for this study was granted by local institutional ethics committee on 1st February 2023 (Application No. 2453/26903/2021). Ethical approval in Ghana was granted by University of Ghana’s College of Health Sciences Ethical and Protocol Review Committee (EPRC) on 22nd May 2023, with renewal granted on 28th June 2024 (Protocol ID No. CHS-Et/M.7–P 4.14/2022–2023).

A reference dataset was used to compare with the population in Ghana. The reference dataset was age-gender matched to the GHA-PD and GHA-CL, and comprised of 50 PwP (UK-PD) and 30 healthy controls (UK-CL) recruited in the UK as a subset of the Mobilize-D Clinical Validation Study (Mikolaizak et al., 2022) and Mobilize-D Extension Study (Health and Social Care REC B. Ref: 23/NI/0046) studies, respectively. Ethical approval was issued by the London-Bloomsbury Research Ethics Committee (all UK sites; Ref: 20/PR/0792). Participants in both studies assessed in Newcastle were included if they could walk four meters independently without walking aids and were able to consent and comply with study procedures. Participants with PD were included if they were aged 18 or over, received a clinical diagnosis of PD in accordance with the MDS criteria (Postuma et al., 2015) and were of a mild-moderate disease stage (Hoehn and Yahr stages I to III) (Hoehn and Yahr, 1967).

### 2.2 Demographic and clinical measures

Participants answered interviewer- and self-administered questionnaires. Demographic information was captured; gender, age (years), mass (kg) and height (centimeters). Clinical information and assessment scores were collected for participants with PD, including the Levodopa Equivalent Daily Dose (LEDD) (Tomlinson et al., 2010), the MDS Unified Parkinson’s Disease-Rating Scale (MDS-UPDRS) part II (patient reported activities of daily living) and III (motor symptom severity) (Goetz et al., 2008) and the Montreal Cognitive Assessment (MoCA) (Nasreddine et al., 2005). MDS-UPDRS assessments were performed during the ON medication state for the UK-PD cohort, while for the GHA-PD cohort, ratings were obtained in mixed medication states (both ON and OFF). Additionally, for participants recruited in Ghana, place of residence was recorded as rural or urban.

### 2.3 Digital mobility assessment

For both cohorts, the 7-day real-world monitoring period reflected participants’ habitual routines, including typical medication states. This is standard practice for real-world monitoring studies and ensures that DMOs capture everyday mobility under natural conditions.

#### 2.3.1 Ghana-based assessment

Each participant was asked to wear an Axivity AX6 wearable device [Axivity Ltd, Newcastle upon Tyne, UK; sampling frequency 100 Hz, triaxial accelerometer range ± 8 g, resolution 1 mg (at ± 8 g); triaxial gyroscope range ± 2,000 degrees per second (dps), resolution: 70 mdps (at ± 2000 dps)], and asked to continue their normal routine. Written informed consent was obtained from the participants prior to fixing sensors after study objectives including data handling and storage were explained. The device captured data across seven consecutive days and was attached over the fifth lumbar vertebra (L5) with a medical grade hydrogel adhesive patch (Linxens, Ängelholm, Sweden) and covered with Hypafix™ bandage. After 7 days, participants removed the device and returned it to the researcher at the clinic or the researcher went back to participants’ homes to pick the sensor. The wearable data were then uploaded to a cloud-based processing platform [eScience central (Hiden et al., 2013)] before being processed by site researchers at Newcastle University. No participants were excluded due to device malfunction or insufficient data quality. Data completeness was assessed by active daytime recording duration, defined as the duration between the first and last detected walking bout within each 24-h day period, with no sleep exclusions. Given the exploratory nature of this study, we did not apply any restriction criteria upon active daytime recording duration per day. All participants met the minimum requirement of three valid monitoring days (Buekers et al., 2025).

#### 2.3.2 Comparison dataset from United Kingdom

The participants from UK followed the same protocol. Twenty-six (52%) of UK-PD wore a DynaPort MoveMonitor (McRoberts, the Hague, Netherlands, sampling frequency 100 Hz, triaxial accelerometer range ± 8 g, resolution 1 mg; triaxial gyroscope range ± 2,000 dps, resolution 70 mdps) and the remaining 24 (48%) wore an Axivity AX6 device. All participants in UK-CL wore an Axivity AX6.

#### 2.3.3 Digital mobility outcomes

Twenty-four DMOs were quantified from the raw wearable device data with the Mobilize-D analytical pipeline, a validated sequence of algorithms that transforms raw sensor signals into mobility metrics. This pipeline has been validated against gold standard (laboratory-grade motion capture) and silver standard (inertial modules, distance sensors and pressure insoles) systems (Kirk et al., 2025; Micó-Amigo et al., 2023). These algorithms were implemented in MATLAB^®^ R2023a (Mathworks, California, United States). The same pipeline was implemented upon the participants in UK-PD via e-Science central, a cloud-based processing platform (Hiden et al., 2013). Although two different wearable devices (Axivity AX6 and DynaPort MoveMonitor) were used within the UK dataset, all data were processed using the Mobilize-D pipeline. Previous validation work within Mobilize-D has demonstrated excellent agreement in derived DMOs and concluded that algorithm performance was not materially affected by device type (Del Din et al., 2025a).

DMOs were estimated at a per-stride and per walking bout (WB) level, with WBs defined as sequences containing at least two consecutive strides of both feet. The WB duration thresholds were selected based upon previous reliability validation and overall relevance to real-world activities. Previous work demonstrating that DMOs capturing from these WBs achieve acceptable reliability (based on ICC > 0.8) within a 7-day recording motivating their inclusion ahead of other WB thresholds (Buekers et al., 2024). Additionally shorter WBs (<30 s) typically occur in indoor contexts (e.g., household activities), while longer WBs (>30 s) are more likely to represent outdoor ambulation (Kirk et al., 2026). The 60-s threshold was included to capture sustained outdoor walking. These thresholds were selected for this study because they have been validated across multiple cohorts, and are based on the duration of WBs rather than population-specific characteristics. Shorter WBs, such as moving between rooms within a household, are a universal feature of daily life, and these thresholds capture the functional distinction between brief, task-oriented movements and longer, destination-directed walking. However, caution must be taken regarding these thresholds as they are not validated in African populations.

### 2.4 Feasibility, usability and acceptability assessment

The feasibility and usability questionnaire developed by Rabinovich et al. (2013) was adapted to measure the acceptability and comfort of the Axivity sensor, respectively (Keogh et al., 2023), for the GHA-PD and GHA-CL cohorts. The questionnaire has two parts: Firstly, a 12-item questionnaire rated on a 5-point scale required participants to rate their experiences wearing the Axivity sensor from 1 (low acceptance) to 5 (high acceptance). The questions focused on whether the device had any technical problems, whether the device was comfortable to wear at night, whether it interfered with their daily activities like bathing and how easy it was to use. Secondly, participants were asked to give the device a single score from 0 to 100 and additional comments on what they liked or disliked about the device. The questionnaires were administered in English or interpreted into participants preferred local language (Twi/Ewe). No written translations of the questionnaires were produced as most Ghanaians cannot read and/or write these local languages except for verbal communication. This is because these languages are only taught at the basic level and not used as a medium for teaching and there is the tendency to lose the ability to read and write them as a person pursues higher education.

### 2.5 Statistical analysis

To address aim 1, DMOs were compared across the four cohorts. The distribution of each outcome was assessed graphically using histograms and normal quantile–quantile (Q-Q) plots. Normally distributed outcomes were analyzed using one-way ANOVA with pairwise comparisons performed using Student’s *t*-tests where appropriate. Outcomes with non-normal distributions were analyzed using the Kruskal-Wallis test, followed by pairwise Mann-Whitney U tests. Within the Ghana cohorts (GHA-PD and GHA-CL), additional subgroup analyses compared participants recruited from rural and urban settings. We also provided *post-hoc* sample size calculations to investigate statistical power of this analysis.

For Aim 2, separate multivariable linear regression models were fitted for each DMO to evaluate differences between the GHA-PD and UK-PD cohorts. The original study design incorporated age- and gender-matched cohorts. Complete-case analysis was performed because several covariates contained missing observations, although the overall level of missingness was low (MoCA: 5%; LEDD: 4%; MDS-UPDRS Parts II and III: 1% each; age, gender, and height: no missing data). All missing observations occurred within the Ghana cohort. Consequently, restricting the analyses to complete cases disrupted the original matched structure by excluding participants from one cohort only. To account for this loss of matching, age and gender were explicitly included in the multivariable regression models, together with height, LEDD, MDS-UPDRS Parts II and III, and MoCA, to adjust for potential residual confounding. Model assumptions were assessed using residual-versus-fitted, normal Q-Q, scale-location, and residual-versus-leverage plots. Homoscedasticity was further evaluated using the Breusch-Pagan test, influential observations were assessed using Cook’s distance, and multicollinearity was examined using variance inflation factors. Diagnostic assessments indicated that no outcome transformations were required.

To further investigate the potential influence of baseline differences between cohorts, a post hoc sensitivity analysis using propensity score matching was performed. A series of clinically motivated 1:1 nearest-neighbor propensity score matching strategies without replacement were explored by sequentially incorporating age, MDS-UPDRS Part II, MDS-UPDRS Part III and MoCA into the propensity score model. Propensity scores were estimated using logistic regression, and a caliper width of 0.2 standard deviations of the logit of the propensity score was applied. The matching procedure, including the 1:1 nearest-neighbor approach, matching without replacement and the specified caliper width, was determined a priori and held constant across the candidate propensity score models. Matching performance was evaluated using absolute standardized mean differences and the number of retained matched pairs. The final covariate specification was selected based on the best compromise between covariate balance and retained sample size, without examination of DMO outcome data (Supplementary Table 1). DMOs were then compared between matched pairs using paired Student’s *t*-tests, thereby accounting for the matched design. The distribution of the within-pair differences was assessed graphically using histograms and normal Q-Q plots, which indicated no substantial departures from normality.

For aim 3, responses to the feasibility, usability and acceptability assessment were descriptively analyzed, reporting the number and proportion of responses for GHA-PD and GHA-CL cohorts.

For the primary unadjusted comparisons, between-group differences were quantified using Cohen’s d with 95% confidence intervals (CIs). For the multivariable regression analyses, adjusted regression coefficients (β) with 95% CIs were reported for all model covariates, with *p*-values reported for the primary exposure (cohort). For the propensity score-matched sensitivity analyses, mean paired differences with 95% CIs were reported. To account for multiple testing across the 24 DMOs, *p*-values were adjusted using the Holm procedure to control the family-wise error rate. All statistical computations were performed using Python and R (version 4.5.0).

## 3 Results

### 3.1 Clinical and demographic characteristics

Participants’ clinical and demographic characteristics are presented in Table 1.

**TABLE 1:** Demographic and clinical information for all cohorts.

	UK-PD	GHA-PD	GHA-CL	UK-CL
Number of participants	50	50	30	30
Gender (Male-Female)	31–19	31–19	19–11	21–9
Age (years)	68.3 (9.7)	67.6 (9.7)	66.6 (8.3)	72.3 (7.3)
Height (cm)	170.1 (10.7)	163.7 (10.3)	163.1 (7.3)	172.4 (9.4)
Mass (kg)	78.3 (16.6)	64.2 (14.7)	65.9 (17.3)	79.6 (18.0)
Body mass index (kg/m^2^)	26.9 (4.7)	24.0 (5.1)	24.7 (6.2)	26.6 (4.7)
Faller history (Y/N)	18/32	19/29	–	6/24
Cognition (MoCA)	26.1 (3.3)	22.7 (4.7)	–	27.9 (1.9)
Time since diagnosis (years)	6.9 (5.8)	5.2 (4.6)	–	–
MDS-UPDRS II	9.3 (5.5)	16.4 (9.6)	–	–
MDS-UPDRS III	31.3 (8.3)	46.4 (22.2)	–	–
LEDD (mg/d)	273.8 (238.6)	187.1 (70.6)	–	–

People with Parkinson’s Disease from Ghana and United Kingdom were denoted as GHA-PD and UK-PD, while corresponding controls were denoted as GHA-CL and UK-CL, respectively. All information was provided as mean and standard deviation, mean (sd), except the number of participants and gender. MDS-UPDRS stands for Movement Disorder Society Unified Parkinson’s Disease Rating Scale, and LEDD stands for Levodopa Equivalent Daily Dose. MoCA stands for Montreal Cognitive Assessment.

All cohorts were predominantly male (≥ 62%). Consistent with the matching strategy, the GHA-PD and UK-PD cohorts were comparable in age and gender. Compared with the UK-PD cohort, the GHA-PD cohort had poorer cognitive performance (MoCA: 22.7 ± 4.7 vs. 26.1 ± 3.3) and greater disease severity (MDS-UPDRS Part II: 16.4 ± 9.6 vs. 9.3 ± 5.5; MDS-UPDRS Part III: 46.4 ± 22.2 vs. 31.3 ± 8.3). Participants in the GHA-PD cohort also had lower body mass index, lower LEDD and a shorter disease duration than those in the UK-PD cohort.

### 3.2 Describing real-world mobility in a population from Ghana: Parkinson’s disease vs. healthy older adults

PwP in Ghana were significantly different from controls in 16 out of 24 DMOs (Figure 1 and Table 2), with large effect sizes (|Cohen’s *d*| > 0.70; Figure 1 and Supplementary Figures 1, 2). In general, they walked less with fewer steps per day (2,153 vs. 10,001, *p* < 0.001, *d* = −1.83 [95% CI: −2.36, −1.29]), had lower daily walking time (0.43 vs. 2.07 h, *p* < 0.001, *d* = −1.94 [95% CI: −2.49, −1.40]), fewer WBs per day (120 vs. 288, *p* < 0.001, *d* = −1.92 [95% CI: −2.46, −1.37]) and shorter WB duration (mean and maximum respectively: 8.0 vs. 10.5 s, *p* < 0.001, *d* = −1.34 [95% CI: −1.84, −0.84]; 24.0 vs. 51.3 s, *p* = 0.012, *d* = −0.81 [95% CI: −1.28, −0.34]). Additionally, those in the GHA-PD group had reduced walking speed (10–30 s: 0.60 vs. 0.70 m/s, *p* < 0.001, *d* = −1.08 [95% CI: −1.56, −0.60]; > 30s: 0.67 vs. 0.83 m/s, *p* < 0.001, *d* = −1.02 [95% CI: −1.51, −0.53]) and stride length (10–30s: 0.82 vs. 0.93 m, *p* < 0.001, *d* = −1.28 [95% CI: −1.77, −0.78]; > 30s: 0.89 vs. 1.03 m, *p* < 0.001, d = −1.05 [95% CI: −1.54, −0.56]) compared to GHA-CL. While the DMOs in the rhythm domain did not significantly differ, two of the DMOs related to variability were significantly higher in PwP than healthy controls in GHA cohorts (stride length WBs > 30s: 18% vs. 13%, *p* = 0.001, *d* = 0.98 [95% CI: 0.50, 1.46]; cadence across all WBs: 15% vs. 13%, *p* = 0.012, *d* = 0.83 [95% CI: 0.36, 1.30]).

**FIGURE 1:**
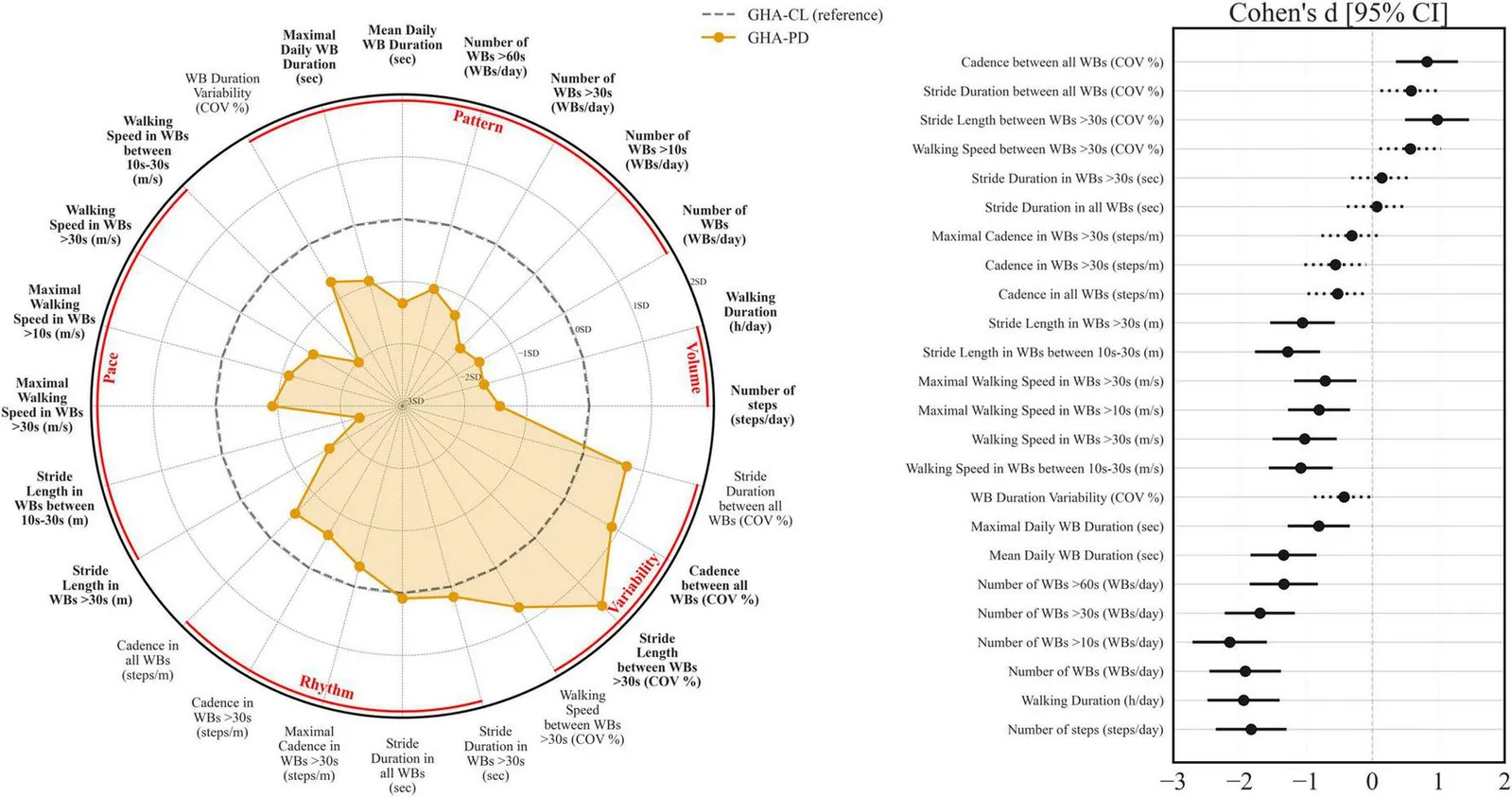
On the left panel, radar plot showing comparisons of digital mobility outcomes between the two populations of people from Ghana. GHA-PD refers to the people with Parkinson’s disease, while GHA-CL is the healthy controls. GHA-CL is the reference cohort that is represented by the central dotted line. Deviation from zero along the axis radiating from the center of the plot represents how many standard deviations (range: −3 to +2 standard deviation (SD), z score based on reference means and SDs) compared group differed from reference. Bolded outcome names denote significant differences (*p* < 0.05). Statistical significance levels can be found in Supplementary Figures 1, 2. Effect sizes, as Cohens’ *d* and 95% confidence interval (CI), are presented in the panel on the right-hand side. Solid lines denote significant differences (*p* < 0.05).

**TABLE 2:** Digital mobility outcomes for people with Parkinson’s Disease (PD) and older adult controls (CL) from Ghana (GHA) and United Kingdom (UK).

	UK-PD	GHA-PD	GHA-CL	UK-CL
Volume				
Number of steps (steps/day)	7,509 (5,815–10,558)	2,153 (1,149–4,255)	10,001 (6,822–14,955)	9,472 (7,266–13,307)
Walking duration (hour/day)	1.29 (1.00–1.66)	0.43 (0.24–0.76)	2.07 (1.37–2.62)	1.61 (1.32–2.25)
Pattern				
Number of WBs (WBs/day)	311 (243–396)	120 (42–157)	288 (249–333)	342 (286–406)
Number of WBs > 10 s (WBs/day)	114 (80–144)	41 (24–64)	156 (113–184)	156 (115–190)
Number of WBs > 30 s (WBs/day)	21 (11–28)	13 (9–19)	55 (32–74)	30 (20–47)
Number of WBs > 60 s (WBs/day)	8 (3–11)	7 (6–9)	18 (12–37)	10 (5–18)
Mean daily WB duration (sec)	7.5 (7.0–8.1)	8.0 (7.3–9.5)	10.5 (9.6–12.2)	8.7 (8.2–9.4)
Maximal daily WB duration (sec)	23.5 (19.8–26.2)	24.0 (20.5–39.8)	51.3 (34.4–68.1)	29.4 (24.2–35.6)
WB duration variability (%)	159 (115–224)	129 (84–174)	187 (157–209)	197 (152–223)
Pace				
Walking speed in WBs between 10 and 30 s (m/s)	0.74 (0.70–0.80)	0.60 (0.49–0.66)	0.70 (0.66–0.73)	0.72 (0.68–0.76)
Walking speed in WBs > 30 s (m/s)	0.91 (0.82–1.02)	0.67 (0.55–0.76)	0.83 (0.74–0.90)	0.94 (0.88–0.99)
Maximal walking speed in WBs > 10 s (m/s)	1.03 (0.95–1.10)	0.87 (0.66–0.97)	1.03 (0.90–1.09)	1.01 (0.95–1.08)
Maximal walking speed in WBs > 30 s (m/s)	1.12 (1.00–1.24)	0.86 (0.72–1.01)	1.05 (0.91–1.11)	1.21 (1.09–1.28)
Stride length in WBs between 10 and 30 s (m)	0.98 (0.91–1.01)	0.82 (0.71–0.89)	0.93 (0.91–0.97)	0.97 (0.93–1.04)
Stride length in WBs > 30 s (m)	1.11 (1.01–1.16)	0.89 (0.80–1.00)	1.03 (0.98–1.08)	1.18 (1.11–1.21)
Rhythm				
Cadence in all WBs (steps/m)	94 (91–95)	88 (85–93)	95 (90–97)	86 (83–88)
Cadence in WBs > 30 s (steps/m)	101 (93–103)	90 (85–94)	96 (91–99)	94 (89–98)
Maximal cadence in WBs > 30 s (steps/m)	109 (102–113)	103 (98–109)	107 (102–109)	106 (99–112)
Stride duration in all WBs (sec)	1.2 (1.2–1.3)	1.3 (1.3–1.4)	1.3 (1.3–1.3)	1.3 (1.2–1.3)
Stride duration in WBs > 30 s (sec)	1.2 (1.1–1.2)	1.3 (1.3–1.4)	1.3 (1.3–1.3)	1.2 (1.2–1.3)
Variability				
Walking speed between WBs > 30 s (%)	19 (16–23)	24 (20–26)	19 (18–23)	21 (19–25)
Stride length between WBs > 30 s (%)	12 (11–16)	18 (15–19)	13 (12–15)	14 (13–16)
Cadence between all WBs (%)	12 (11–12)	15 (13–16)	13 (10–15)	12 (11–13)
Stride duration between all WBs (%)	17 (15–18)	18 (17–20)	17 (16–19)	14 (14–15)

Values are presented as median and the interquartile range. Walking bout (WB) is defined as a sequence containing at least two consecutive strides of both feet.

As shown in Figure 2, place of residence in Ghana primarily influenced walking characteristics among PwP. Specifically, participants from rural settings exhibited a greater number of WBs greater than 30 s and 60 s, as well as longer mean and maximal daily WB durations compared to those from urban settings; however, these differences did not reach statistical significance after correcting *p* values for multiple testing. *Post-hoc* power calculations for the rural-urban subgroup analyses (Rural-GHA-PD: *n* = 25, Urban-GHA-PD: *n* = 25) indicated 40% power to detect a medium effect (*d* = 0.50) and 68% power to detect a large effect (*d* = 0.80) at α = 0.05. For the control subgroup (Rural-GHA-CL: *n* = 19, Urban-GHA-CL: *n* = 11), power was substantially lower.

**FIGURE 2:**
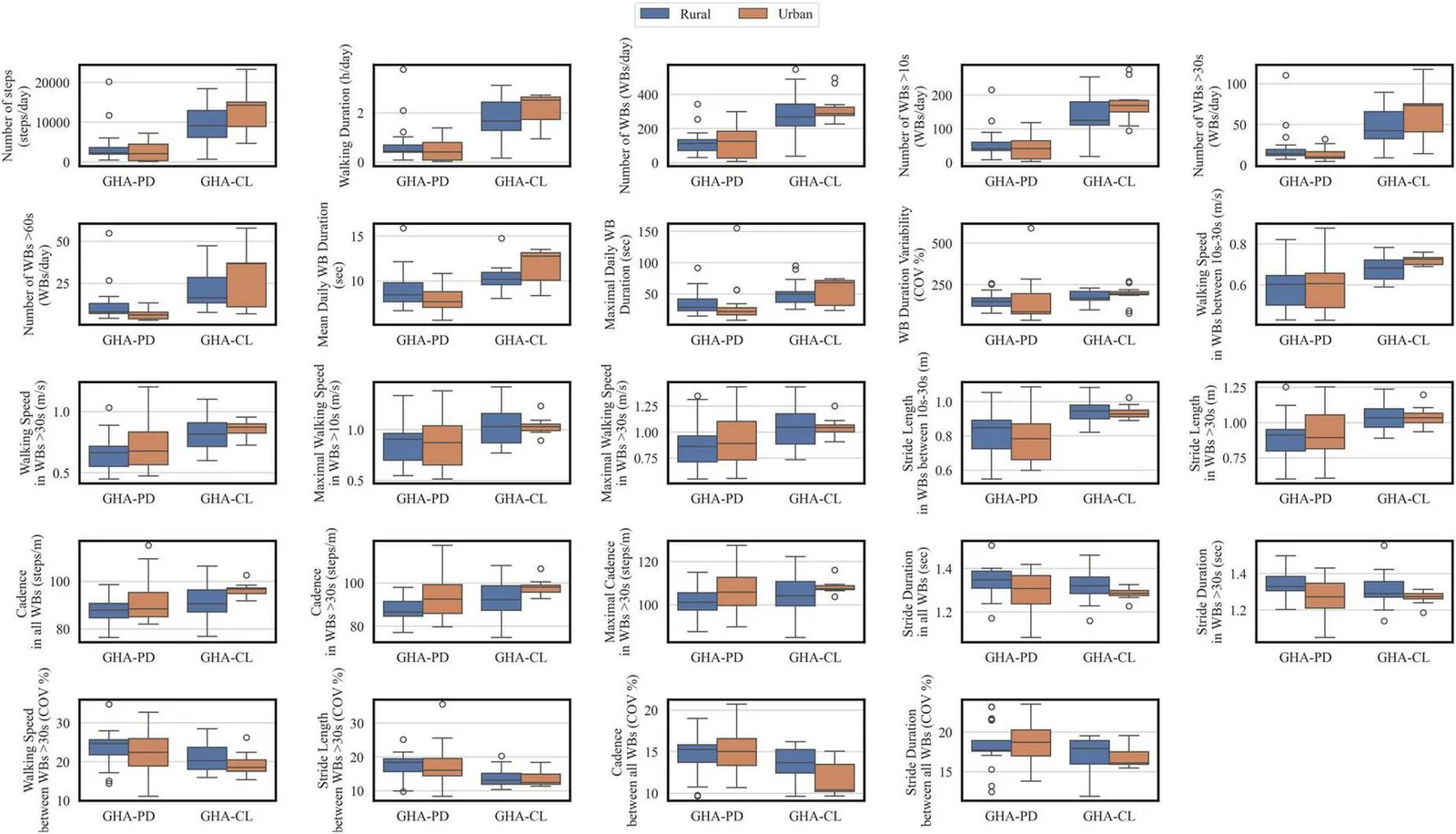
Digital mobility outcomes for rural (blue) vs. urban (orange) boxplots and comparisons within the people with Parkinson’s disease from Ghana (GHA-PD) and the healthy controls (GHA-CL). Statistically significant differences, if any, are presented above each cohort.

### 3.3 Exploring context in two geographically distant cohorts of people with Parkinson’s disease: Ghana vs. United Kingdom

Unadjusted comparisons showed that participants in the GHA-PD cohort differed significantly from the UK-PD cohort in 17 of the 24 DMOs, with moderate-to-large effect sizes (|Cohen’s d| ≥ 0.65; Table 2, Figure 3, and Supplementary Figures 1, 2). Significant between-cohort differences were observed across measures of walking volume, walking pattern, pace, rhythm and variability. In contrast, no significant differences were observed for mean daily WB duration, maximal daily WB duration, WB duration variability, the number of WBs lasting > 30 s or > 60 s, maximal cadence during WBs > 30 s, or cadence across all WBs.

**FIGURE 3:**
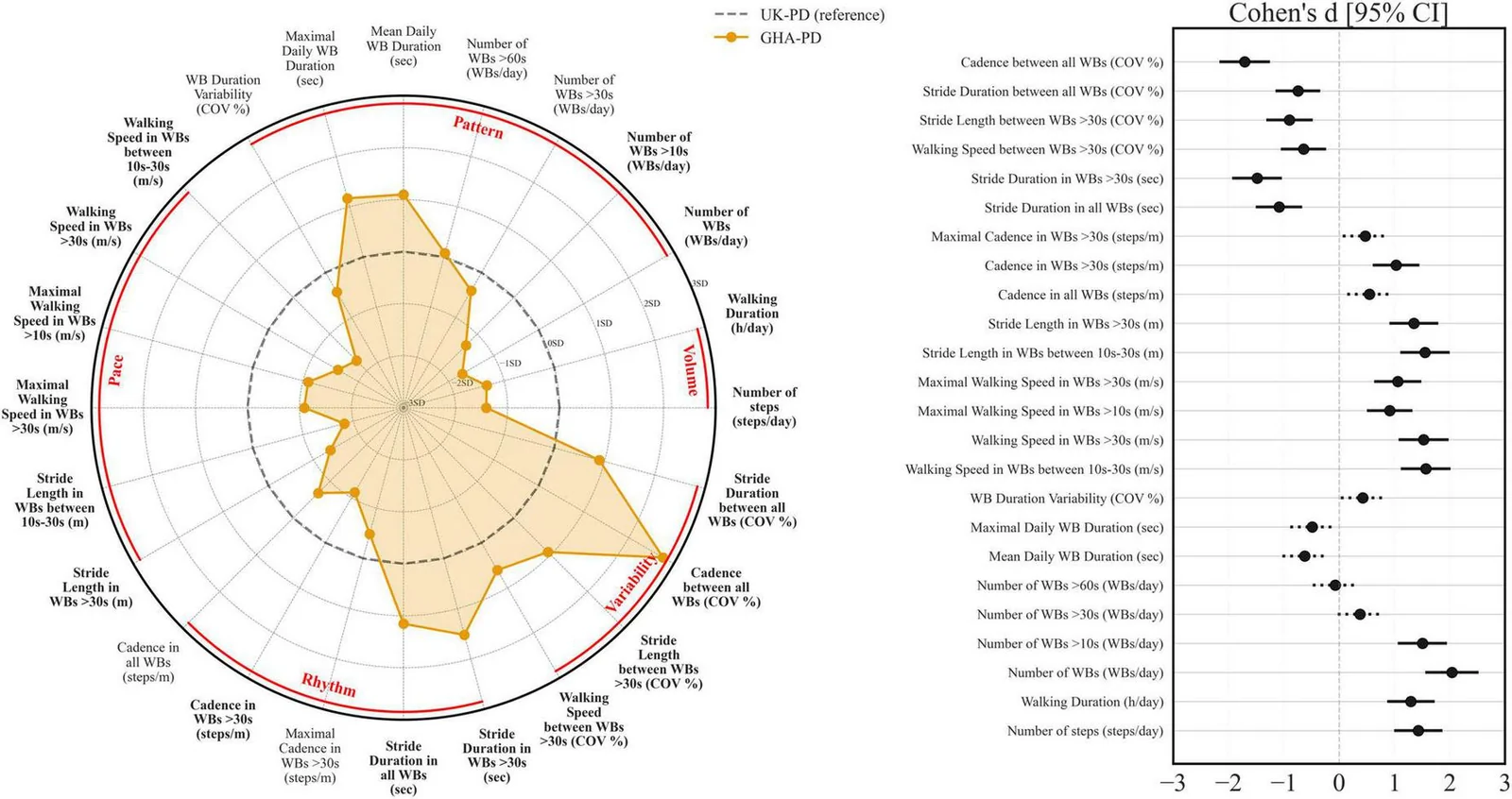
On the left panel, radar plot showing comparisons of digital mobility outcomes between the two populations of people with Parkinson’s disease (PD) from Ghana (GHA-PD) and UK (UK-PD). UK-PD is the reference cohort that is represented by the central dotted line. Deviation from zero along the axis radiating from the center of the plot represents how many standard deviations (range: ± 3 standard deviation (SD), z score based on reference means and SDs) compared group differed from reference. Bolded outcome names denote significant differences (*p* < 0.05). Statistical significance levels can be found in Supplementary Figures 1, 2. Effect sizes, as Cohens’ *d* and 95% confidence interval (CI), are presented in the panel on the right-hand side. Solid lines denote significant differences (*p* < 0.05).

After adjustment for age, gender, height, LEDD, MDS-UPDRS Parts II and III, and MoCA, evidence of between-cohort differences existed for 18 of the 24 DMOs following Holm correction for multiple testing (Table 3). Compared with the UK-PD cohort, the GHA-PD cohort took, on average, 3,340 fewer daily steps per day (β = −3340, 95% CI −5002 to −1679) and completed 174 fewer daily WBs per day (β = −174, 95% CI −227 to −123). The GHA-PD cohort also walked more slowly, with lower walking speed during WBs lasting 10–30 s (β = −0.14 m/s, 95% CI −0.18 to −0.09) and > 30 s (β = −0.22 m/s, 95% CI −0.30 to −0.14), accompanied by shorter stride length across both WB durations. In addition, cadence during WBs lasting > 30 s was lower in the GHA-PD cohort (β = −9 steps/min, 95% CI −13 to −5), while cadence variability was higher (β = 3%, 95% CI 2–4%). Compared with the unadjusted analyses, evidence of between-cohort differences emerged for mean daily and maximal daily WB duration after adjustment, whereas stride duration variability was no longer statistically significant following Holm correction. No evidence of between-cohort differences was observed for the number of WBs lasting > 30 s or > 60 s, cadence across all walking bouts, maximal cadence during WBs > 30 s, or WB duration variability in either the unadjusted or adjusted analyses.

**TABLE 3:** Association between digital mobility outcomes and cohort from multivariable linear regression models adjusted for age, gender, height, Levodopa Equivalent Dose (LEDD), Movement Disorders Society Unified Parkinson’s Disease-Rating Scale (MDS-UPDRS) Part II and III, and Montreal Cognitive Assessment (MoCA).

	Ghana vs. UK-PD, *p*-value	Age (years)	Gender	Height (cm)	LEDD (mg/day)	MDS-UPDRS Part II	MDS-UPDRS Part III	MoCA
Volume								
Number of steps (steps/day) 93	**−3340 (**−**5002,–1679), 0.002**	**−110 (**−**177,-42)**	488 (**−**1195, 2172)	**−**18 (**−**103,66)	2 (**−**2,5)	**−**86 (**−**177, 6)	**−**43 (**−**85, 0)	105 (**−**84, 294)
Walking duration (hour/day)	**−0.47 (−0.76, −0.18), 0.018**	**−0.02 (−0.03,** −**0.01)**	0.10 (−0.19, 0.40)	0.00 (**−**0.02, 0.01)	0.00 (**−**0.00, 0.00)	**−0.02 (−0.03, −0.00)**	**−0.01 (−0.02, −0.00)**	0.02 (**−**0.01, 0.05)
Pattern								
Number of WBs (WBs/day)	**−174 (−227, −123), < 0.001**	**−4 (−6, −2)**	16 (**−**4, 69)	**−**0 (**−**3, 2)	**0 (0, 0)**	**−3 (−6, −0)**	**−**1 (**−**2, 0)	**−**0 (**−**6, 6)
Number of WBs > 10 s (WBs/day)	**−51 (−73, −28), < 0.001**	**−2 (−3, −1)**	16 (**−**7, 38)	**−**0 (**−**1, 1)	0 (**−**0, 0)	**−**1 (**−**2, 0)	**−**1 [**−**1, 0)	1 (**−**2, 3)
Number of WBs > 30 s (WBs/day)	0 (**−**7, 8), 1.000	**−0 (−1, −0)**	5 (**−**3, 13)	**−**0 (**−**0, 0)	0 (**−**0, 0)	**−**0 (**−**1, 0)	**−0 (−0, −0)**	1 (**−**0, 2)
Number of WBs > 60 s (WBs/day)	3 (**−**2, 7), 0.670	**−**0 (**−**0, 0)	1 (**−**3, 6)	**−**0 (**−**0, 0)	0 (**−**0, 0)	**−**0 (**−**0, 0)	**−0 (−0, −0)**	0 (**−**0, 0)
Mean daily WB duration (sec)	**1.09 (0.37, 1.81), 0.029**	**−**0.00 (**−**0.03, 0.03)	0.64 (**−**0.09, 1.37)	**−**0.00 (**−**0.04, 0.04)	0.00 (**−**0.00, 0.00)	**−**0.01 (**−**0.05, 0.03)	**−**0.01 (**−**0.03, 0.01)	0.05 (**−**0.03, 0.13)
Maximal daily WB duration (sec)	**17.87 (9.02, 26.71), 0.001**	0.00 (**−**0.36, 0.36)	1.78 (**−**7.18, 10.74)	0.11 (**−**0.34, 0.56)	0.00 (**−**0.02, 0.02)	**−0.58 (−1.07, −0.09)**	**−**0.19 (**−**0.42, 0.04)	0.90 (**−**0.11, 1.91)
WB duration variability (%)	**−**6 (**−**52, 40), 1.000	**−**0 (**−**2, 2)	**−**17 (**−**64, 31)	1 (**−**1, 3)	0 (**−**0, 0)	**−**2 (**−**4, 1)	**−**1 (**−**2, 1)	4 (**−**2, 9)
Pace								
Walking speed in WBs between 10 and 30 s (m/s)	**−0.14 (−0.18, −0.09), < 0.001**	**−0.00 (−0.01, −0.00)**	**−**0.00 (**−**0.01, 0.00)	0.00 (**−**0.00, 0.00)	0.00 (**−**0.00, 0.00)	**−**0.00 (**−**0.00, 0.00)	**−**0.00 (**−**0.00, 0.00)	0.00 (**−**0.00, 0.01)
Walking speed in WBs > 30 s (m/s)	**−0.22 (−0.30, −0.14), < 0.001**	**−0.01 (−0.01, −0.00)**	−0.00 (−0.08, 0.08)	0.00 (**−**0.00, 0.00)	**−**0.00 (**−**0.00, 0.00)	**−**0.00 (**−**0.00, 0.00)	**−**0.00 (**−**0.00, 0.00)	0.00 (**−**0.01, 0.01)
Maximal walking speed in WBs > 10 s (m/s)	−**0.13 (**−**0.23,** −**0.04), 0.036**	−**0.01 (**−**0.01,** −**0.00)**	0.03 (−0.07, 0.12)	0.00 (**−**0.00, 0.00)	**−**0.00 (**−**0.00, 0.00)	**−**0.00 (**−**0.00, 0.00)	**−**0.00 (**−**0.00, 0.00)	0.00 (**−**0.00, 0.01)
Maximal Walking Speed in WBs > 30 s (m/s)	−**0.18 (**−**0.28,** −**0.08), 0.008**	−**0.01 (**−**0.01,** −**0.01)**	0.01 (−0.09, 0.11)	0.00 (**−**0.00, 0.01)	0.00 (**−**0.00, 0.00)	**−**0.00 (**−**0.01, 0.00)	**−**0.00 (**−**0.0, 0.00)	0.00 (**−**0.01, 0.02)
Stride length in WBs between 10 and 30 s (m)	−**0.13 (**−**0.18,** −**0.08), < 0.001**	−**0.00 (**−**0.01,** −**0.00)**	0.01 (−0.04, 0.07)]	0.00 (**−**0.00, 0.00)	0.00 (**−**0.00, 0.00)	**−**0.00 (**−**0.00, 0.00)	**−**0.00 (**−**0.00, 0.00)	0.00 (**−**0.00, 0.01)
Stride length in WBs > 30 s (m)	−**0.16 (**−**0.24,** −**0.09), 0.001**	−**0.01 (**−**0.01,** −**0.00)**	0.01 (−0.07, 0.09)	0.00 (**−**0.00, 0.01)	0.00 (**−**0.00, 0.00)	**−**0.00 (**−**0.01, 0.00)	**−**0.00 (**−**0.00, 0.00)	0.00 (**−**0.01, 0.01)
Rhythm								
Cadence in all WBs (steps/m)	−**4 (**−**7,** −**0), 0.136**	−**0 (**−**0,** −**0)**	−3 (−6, 0)	**−**0 (**−**0, 0)	0 (**−**0, 0)	**−**0 (**−**0, 0)	0 (**−**0, 0)	**−**0 (**−**0, 0)
Cadence in WBs > 30 s (steps/m)	−**9 (**−**13,** −**5), 0.001**	−**0 (**−**0,** −**0)**	−3 (−7, 2)	**−**0 (**−**0, 0)	**−**0 (**−**0, 0)	**−**0 (**−**0, 0)	**−**0 (**−**0, 0)	**−**0 (**−**1, 0)
Maximal cadence in WBs > 30 s (steps/m)	−4 (−9, 0), 0.216	−**0 (**−**0,** −**0)**	−2 (−7, 2)	−0 (−0, 0)	−0 (−0, 0)	−0 (−0, 0)	−0 (−0, 0)	−0 (−1, 0)
Stride duration in all WBs (sec)	**0.09 (0.05, 0.14), < 0.001**	**0.00 (0.00, 0.00)**	0.03 (−0.01, 0.07)	0.00 (−0.00, 0.00)	−0.00 (−0.00, 0.00)	−0.00 (−0.00, 0.00)	0.00 (−0.00, 0.00)	0.00 (−0.00, 0.01)
Stride duration in WBs > 30 s (sec)	**0.15 (0.10, 0.20), < 0.001**	**0.00 (0.00, 0.00)**	0.00 (−0.02, 0.08)	0.00 (−0.00, 0.00)	0.00 (−0.00, 0.00)	−0.00 (−0.00, 0.00)	0.00 (−0.00, 0.00)	0.00 (−0.00, 0.01)
Variability								
Walking speed between WBs > 30 s (%)	**4 (1, 7), 0.018**	**−0 (−0, −0)**	2 (**−**1, 4)	**−**0 (**−**0, 0)	0 (**−**0, 0)	**−**0 (**−**0, 0)	0 (**−**0, 0)	0 (**−**0, 0)
Stride length between WBs > 30 s (%)	**5 (2, 7), 0.004**	**−0 (−0, −0)**	1 (**−**2, 3)	0 (**−**0, 0)	0 (**−**0, 0)	**−**0 (−0, 0)	0 (−0, 0)	0 (−0, 0)
Cadence between all WBs (%)	**3 (2, 4), < 0.001**	−0 (−0, 0)	−0 (−1, 1)	−0 (−0, 0)	−0 (−0, 0)	**0 (0, 0)**	−0 (−0, 0)	−0 (−0, 0)
Stride duration between all WBs (%)	**1 (0, 2), 0.111**	−0 (−0, 0)	−0 (−1, 1)	−0 (−0, 0)	0 (−0, 0)	**0 (0, 0)**	−**0 (**−**0,** −**0)**	−0 (−0, 0)

Results are presented as regression coefficients (β), 95% confidence intervals (CI), and Holm-adjusted *p*-values for the cohort effect. The reference categories were cohort of people with Parkinson’s disease in UK (UK-PD) for cohort and female for gender. Positive cohort coefficients indicate higher values in the Ghana cohort (GHA-PD) than in UK-PD. Walking bout (WB) is defined as a sequence containing at least two consecutive strides of both feet. Negative signs appearing with zero (e.g., − 0) are artifacts of rounding values that are near-zero at the reported level of significance. Bold font indicates 95% confidence intervals that do not include zero. Bold values indicate statistical significance.

The propensity score-matched sensitivity analysis produced effect estimates that were broadly consistent with those from the primary adjusted analyses (Supplementary Table 2). The direction and magnitude of the between-cohort differences remained largely unchanged for the principal digital mobility outcomes, including number steps and WBs per day, walking speed, stride length, cadence during longer WBs and stride duration. Confidence intervals were wider and fewer outcomes remained statistically significant following Holm correction because matching reduced the analytical sample to 20 matched pairs. Overall, the sensitivity analysis supported the robustness of the primary findings.

In contrast, the controls (GHA-CL vs. UK-CL) showed broadly comparable DMOs, particularly for number of steps and walking duration per day, suggesting similar levels of walking activity (Supplementary Figures 3–5). Additionally, comparison within the UK cohort (UK-CL vs. UK-PD) revealed three statistically significant DMOs out of 24 (mean daily WB duration, cadence and stride duration in all WBs), indicating relatively modest mobility differences between healthy controls and PwP in the UK.

Associations between the adjustment covariates and the DMOs were generally modest and limited to a subset of outcomes (Table 3). Among the adjustment covariates, age exhibited the most consistent pattern of non-zero regression coefficients across the DMOs. In contrast, the 95% CI for gender, height and MoCA consistently included zero, suggesting little evidence of independent associations after adjustment. MDS-UPDRS Part II and III showed non-zero associations with a small number of outcomes, whereas the estimated effects of LEDD were limited to the number of daily WBs. Overall, the associations with the adjustment covariates were generally weaker and less consistent than the between-cohort differences.

### 3.4 Feasibility, usability and acceptability of digital data collection

GHA-PD and GHA-CL both had a median active daytime recording duration of 7 days (maximum duration), with all wearing the device more than the minimum recommended 3 days. On average people had 12.14 h per day of active daytime recording duration.

As presented in Figure 4, feasibility was high, with 100% of participants in both cohorts reporting no technical problems with the wearable sensor. Usability was generally favorable: 83% of GHA-PD and 70% of GHA-CL responses were positive, with all participants in both groups finding it easy to get started with the wearable device. The most frequent usability issue was taking off the device, which the GHA-CL group had more difficulty with (22% unfavorable in GHA-PD; 66% unfavorable in GHA-CL). Acceptability showed more mixed responses: 61% favorable in GHA-PD and 65% unfavorable in GHA-CL. Most participants did not feel embarrassed (96% GHA-PD; 93% GHA-CL), but discomfort (92% GHA-PD; 79% GHA-CL) and perceived privacy invasion (96% GHA-PD; 83% GHA-CL) were common concerns. Participant privacy was prioritized, and only the lower back was exposed for sensor placement in a private room. Nonetheless, some participants reported that attaching a foreign object to their lower back felt intrusive.

**FIGURE 4:**
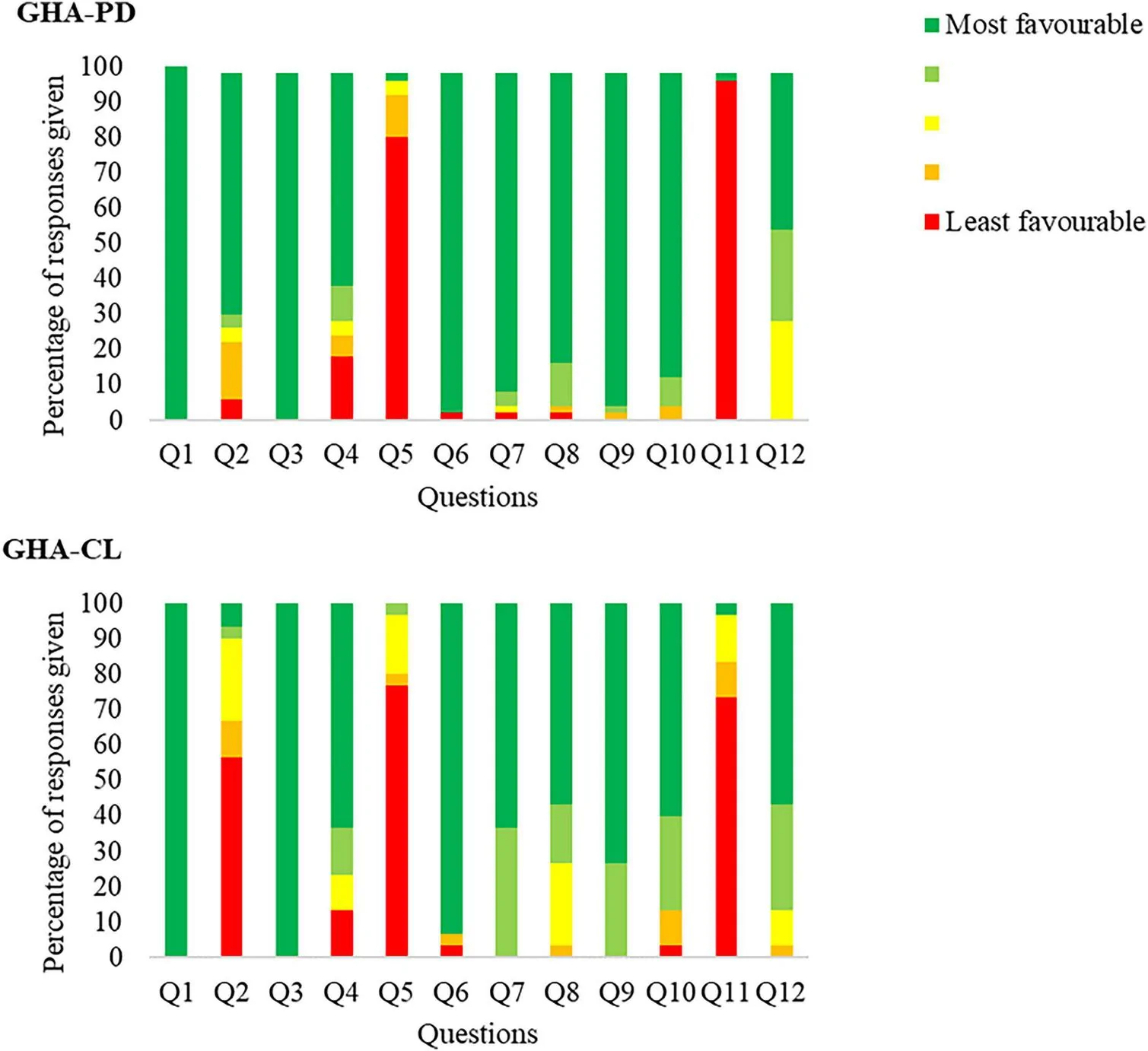
Responses given to the usability questionnaire (%) for participants recruited in Ghana. Q1: How much trouble did you have getting started with the wearable device? Q2: The wearable device was easy to put on/take off. Q3: I experienced technical problems with the wearable technology system. Q4: The wearable device interfered with my normal activities. Q5: I felt comfortable wearing the wearable device. Q6: I felt embarrassed wearing the wearable device. Q7: The instructions on how to use the wearable device were clear. Q8: Using the wearable device on a daily basis was easy. Q9: The wearable device was bulky/heavy. Q10: The wearable device bothered me in bed. Q11: I felt my privacy was invaded by the wearable device. Q12: If my doctor would like to use the wearable device to assess my activity I would be willing to wear it and use it for. Color code from red (score = 1 for least favorable response) to green (score = 5 for most favorable response).

## 4 Discussion

This study is the first study investigating real-world mobility in a population of PwP from Africa (Walker et al., 2023). Our preliminary findings suggest that that mobility patterns in this West African cohort may differ from those observed in UK counterparts, with trends toward lower activity levels and more impaired gait outcomes, even when accounting for clinical and demographic differences. While the study demonstrated excellent active daytime recording duration and high usability scores, some participants reported challenges regarding device comfort and privacy concerns. These preliminary observations are discussed below.

### 4.1 Real-world mobility in a population of people from Ghana: Parkison’s disease vs. older adults

While no study has characterized walking volume in PwP, specifically in Ghana or Africa as a whole, our preliminary observations show that GHA-PD recorded a median of 2,153 steps per day and 120 bouts per day, less than half the steps and bouts of GHA-CL. The deviations from the controls were significant and supported by large effect sizes. These observed patterns appear consistent with studies of other mobility-limiting conditions in Africa that report sedentary lifestyles. For example, approximately 80% of stroke survivors in similar contexts are reported to be physically inactive, with fewer than 7,100 daily steps (Honado et al., 2023), whereas 94% of our GHA-PD cohort fell below this threshold. Furthermore, findings from South African studies using wearable technology have noted that stroke survivors and older adults with diabetes average approximately 3,000 steps per day (Joseph et al., 2020; Siddiqui et al., 2018), comparable to the median 3,106 daily steps observed in our GHA-PD cohort, despite differences in outcome calculation methods. The lower activity levels observed in older adults with disabilities in this population may be influenced by multiple factors. Environmental barriers (e.g., poor walkability, uneven terrain) and safety concerns (e.g., fear of falls, crime) may restrict outdoor activity. Sociocultural factors, including social stigma leading to isolation (Fothergill-Misbah, 2023), and cultural norms involving extended family care system (Adonteng-Kissi et al., 2022), may further influence activity engagement.

In this cohort, the observed differences in walking volume between GHA-PD and GHA-CL align with patterns of functional impairment associated with PD seen in European populations (Lord et al., 2013; Mirelman et al., 2024). Specifically, metrics within the pace domain, including walking speed, stride length and cadence, were lower in GHA-PD. While stride duration was similar between groups, gait variability (e.g., stride length and cadence variability) was higher in GHA-PD compared to GHA-CL, which may reflect differences in motor control and consistency (Hausdorff, 2005).

Subgroup analyses on the environment (urban vs. rural within Ghana) revealed no significant differences between groups. However, numerical trends were observed in the GHA-PD cohort; specifically, rural participants exhibited longer WBs ( > 30 s and > 60 s) alongside a longer mean daily WB duration compared to urban participants. This pattern of increased activity in rural settings aligns with observations in other African (Cook, 2022; Kirunda et al., 2016) and European (Ringsberg et al., 1998) cohorts. While these trends were more pronounced in the PD group than in controls, the lack of statistical significance suggests that residential environment may not be a primary driver of mobility differences in this specific study population. *Post-hoc* power calculations indicated that the subgroup analyses were underpowered to detect moderate effects, particularly given the modest sample sizes. These findings should therefore be considered exploratory and require confirmation in larger, adequately powered studies.

### 4.2 Context in geographically distant cohorts of people: Ghana vs. United Kingdom

Analysis of the DMOs revealed significant cross-cohort divergences in mobility. While GHA-CL mobility levels were comparable to UK-CL, GHA-PD exhibited significantly lower mobility than UK-PD across multiple domains, with large effect sizes. The modest within-UK differences likely reflect mild disease severity and survivorship bias favoring ambulatory participants, but do not undermine DMO sensitivity as the same metrics robustly distinguished GHA-PD from GHA-CL (16/24) and UK-PD (17/24).

Walking volumes were comparable between the control cohorts. While UK-PD exhibited a downward trend in walking activity compared to the UK-CL group, this difference did not reach statistical significance. In contrast, a significant reduction in walking volume was observed in GHA-PD relative to their age- and gender-matched UK-PD counterparts, even after adjustment for height, LEDD, MDS-UPDRS parts II, III and MoCA. Additionally, the analysis by subsampling PD cohorts to match disease severity and cognition included only 14 participants from both PD cohorts. Noting the decreased statistical power due to lower number of participants involved, the cohort still had significant associations with the DMOs, achieving comparable effect sizes, given no significant differences between the clinical and demographic measures between the two subsamples, though this finding should be interpreted cautiously due to reduced power. The observed differences might be influenced by differences in methodological variations (e.g., real-world data-capture conditions, cohort composition).

Regarding walking patterns, GHA-CL showed comparable or greater engagement in longer WBs ( > 30 s and > 60 s) than UK-CL, but the opposite trend was observed between the PD cohorts, with GHA-PD having fewer short bouts than UK-PD. Similar to walking volume, the differences between the UK-CL and UK-PD were not significant. These contrasts suggest that the lower walking volume in GHA-PD is unlikely to reflect a general population-level difference in activity between the two countries. The observation of fewer shorter walking bouts in GHA-PD compared to UK-PD may suggest a potential reduction in independence for everyday tasks. This could reflect a context where PwP in Ghana are less encouraged to maintain activities typically completed during short WBs (e.g., walking to the kitchen).

With respect to the pace domain, the fundamental mechanics of gait also appeared to differ between the groups. GHA-PD had slower walking speed and shorter stride lengths than all other groups. For context, walking speed during longer WBs in control cohorts was within ranges observed internationally (ranging from 0.52 to 1.09 m/s) (Sprague et al., 2023). Although the difference between the two control groups was statistically significant (Supplementary Figure 4), it might depend on other factors such as the general wellbeing of the older cohorts. Evidence from studies involving older populations has shown that a Tanzanian cohort with poorer general health walked slower than healthier Australians, while similarly healthy Nigerians walked even faster than both (Sprague et al., 2023). This highlights the influence of overall health on walking speed rather than contextual factors alone.

In the rhythm domain, GHA-PD exhibited longer stride duration (in WBs > 30 s and all WBs) and higher cadence (in WBs > 30 s) than UK-PD, despite their slower walking speed. Similarly, GHA-CL showed longer stride duration (WBs > 30 s) and higher cadence (all WBs) than UK-CL. No rhythm differences were observed between GHA-CL and GHA-PD. The apparent dissociation between slower speed yet longer stride duration and higher cadence in GHA-PD may reflect a compensatory adaptation to maintain stability.

For gait variability, despite the similarities between the older adult cohorts from Ghana and the UK (Supplementary Figure 5), GHA-PD exhibited significantly greater variability than UK-PD across stride length, cadence, and walking speed. This finding suggests that mobility is not only reduced in quantity but also less stable and consistent, potentially reflecting more advanced or less effectively managed disease states (Lang et al., 2024; Rochester et al., 2014). Importantly, this greater variability was not solely attributable to bradykinesia (Frenkel-Toledo et al., 2005), highlighting the complexity of motor control impairments in PD. Greater gait variability may contribute to further deterioration, as it has been associated with an increased risk of falls (Ma et al., 2022). However, the finding regarding the gait variability should be treated cautiously as these DMOs achieved low specificity (Mancini et al., 2025), especially with shorter WB durations (Galna et al., 2013).

The observed between-country differences are likely multifactorial. First, differences in disease severity between cohorts may directly influence mobility performance. An important consideration is the difference in medication-state assessment between cohorts. UK-PD were assessed during the ON medication state, whereas GHA-PD were assessed in mixed ON/OFF states. However, the real-world DMO data were collected during habitual daily routines in both cohorts, capturing typical medication states and everyday mobility under natural conditions. Nevertheless, the lower mobility and greater gait variability observed in GHA-PD may partly reflect suboptimal medication management or greater OFF-time burden, as DMOs are sensitive to dopaminergic status (Packer et al., 2025). This may be compounded by differences in available treatments. In the UK, additional medications are available to reduce OFF-time and manage fluctuations, whereas treatment options in sub-Saharan Africa are typically limited to levodopa and anticholinergics (Makanjuola et al., 2021). Second, healthcare-system factors, including access to specialist care, rehabilitation services, and clinical management pathways, may affect functional outcomes (Dotchin and Walker, 2012; Williams et al., 2018). Third, environmental characteristics, such as neighborhood walkability, transport infrastructure, and climate, may impact on opportunities for everyday mobility. Finally, sociocultural factors, including lifestyle, social participation, cultural expectations, and behavioral norms, may shape how individuals engage in daily activities (Adonteng-Kissi et al., 2022; Dotchin et al., 2014). These influences are conceptually distinct and should be considered separately when interpreting international differences in real-world mobility in PD.

### 4.3 Associations between DMOs and confounding factors

We adjusted for age, gender, height, LEDD, and MDS-UPDRS parts II, III and MoCA to investigate whether the observed differences in DMOs between the GHA-PD and UK-PD cohorts could be explained by these demographic and clinical characteristics. After adjustment using multivariable linear regression models, the between-cohort differences remained evident across several DMO domains, whereas the measured clinical and demographic factors showed comparatively limited independent associations. Furthermore, the propensity score-matched sensitivity analysis yielded effect estimates that were broadly consistent with those from the primary adjusted analyses, supporting the robustness of the observed between-cohort differences. However, some residual imbalance remained in the matched sample, particularly for LEDD and, to a lesser extent, height. As the matched analysis was based on paired comparisons and the reduced sample size precluded further multivariable adjustment, some of the observed differences in this sensitivity analysis may reflect residual confounding by these factors. Among the adjustment covariates, age exhibited the most consistent pattern of associations across the DMOs. In contrast, gender, height and MoCA showed little evidence of independent associations after adjustment. MDS-UPDRS Parts II and III were associated with only a small subset of the DMOs, whereas the estimated effects of LEDD were limited primarily to the number of WBs per day. Although LEDD demonstrated only limited independent associations in the adjusted models, the numerically lower values observed in the GHA-PD cohort may still be clinically relevant, as reduced dopaminergic treatment intensity could contribute to poorer real-world mobility.

These findings align with previous research (Galperin et al., 2019; Mirelman et al., 2024) indicating that clinical scores do not fully explain real-world DMOs in PD cohorts. One possible reason is that the MDS-UPDRS focuses on signs and symptoms assessed in clinical settings, whereas DMOs capture functional performance during daily life, which includes variability due to behavioral context, environmental complexity, and motor fluctuations over longer periods than can be measured in a clinical or research setting. Consequently, real-world DMOs may provide complementary information to standard clinical assessments and could help capture motor fluctuations related to on/off medication states.

Taken together, the persistence of the between-cohort differences after multivariable adjustment and in the propensity score-matched sensitivity analysis suggests that geographical, clinical, healthcare-related, environmental, and sociocultural influences, may contribute to the observed DMO differences between the two PD cohorts, beyond the measured demographic and clinical characteristics alone.

### 4.4 Feasibility, usability and acceptability of digital data collection

Excellent feasibility was demonstrated for both PD and control groups completing the real-world wearable data collection, with 97% achieving the recommended 7 days of continuous monitoring for reliable DMO capture, as suggested by Buekers et al. (2024). This compliance is higher than the 91% valid data reported in a Tanzanian study of older adults (Del Din et al., 2025b). All participants reported no technical issues, further supporting feasibility.

Usability was high in GHA-PD (83% favorable responses), similar to findings in Western PD populations (Debelle et al., 2023), but lower in GHA-CL, mainly due to difficulty removing the device. This may be linked to the device’s adhesive attachment method, affixed with a hydrocolloid-adhesive patch and covered with medical tape, making removal and reattachment less straightforward, and to participants with PD receiving more help from carers or family members when re-equipping the device. Future studies could provide similar support to control participants and adapt usability scales to capture assistance received.

Acceptability was generally favorable, though many participants reported discomfort, contrasting with high comfort levels reported in European PD populations (Debelle et al., 2023). Participants of the Tanzanian feasibility study reported itching at the sticker site (Del Din et al., 2025b), thus climate-related factors (heat, humidity) likely contributed, with reports of rashes, itching, sweating and general irritation which was more consistently reported by the GHA-CL, suggesting the need for attachment options better suited to warmer environments. Additionally, unfamiliarity with wearing the device could also contribute to discomfort. Another study on European participants using a Velcro-attached McRoberts Dynaport MM+ (Keogh et al., 2023), found high levels of comfort, though some still reported discomfort with the Velcro waist strap. This implies that personal preference may play a role, and offering different attachment options could provide advantages facilitating widespread deployment. Privacy concerns were also common, potentially reflecting broader digital health equity and trust issues in sub-Saharan Africa (Masinde et al., 2025; Qoseem et al., 2024). Education on data handling and safeguards could improve acceptability. Overall, digital data collection with the wearable device may be feasible and usable, but comfort and privacy warrant further attention.

### 4.5 Clinical implications and future directions

The low mobility levels observed in the GHA-PD cohort may reflect limited access to PD-specific care, lack of physiotherapy services or low social recognition of PD, as similarly reported by previous works on similar contexts (Akinyemi, 2012; Williams et al., 2018) although these aspects vary within and between populations and countries. Furthermore, the observed differences in mobility patterns suggest that the lived experience of aging and health may vary across regions or countries. This highlights the importance of conducting qualitative research alongside quantitative research to aid the interpretation of quantitative findings, especially regarding aspects such as stigma, the environment, and cultural expectations. More fundamentally, however, DMOs can only be useful once individuals have accessed clinical services; foundational barriers of low awareness, underdiagnosis, and limited healthcare access must first be addressed.

Building on these considerations, future research could investigate contextual lifestyle factors, such as physical activity norms, environmental walkability, access to transportation and health system capacity, to understand how these shape mobility outcomes. Finally, associated risks of less and poor-quality gait characteristics require further investigations of their impact on falls and reduced independence.

### 4.6 Limitations

This study has some limitations. First, residual confounding from unmeasured factors (e.g., nutrition, comorbidities, habitual physical activity, environmental factors, socioeconomic context, social support and depressive symptoms) likely remains. Psychosocial state and wellbeing in particular have been shown to influence habitual physical activity in a UK-based population of PwP (McArdle et al., 2022). We attempted to mitigate this by adjusting for key known variables, but future studies should consider broader contextual and clinical matching strategies and include formal measures of mood and social determinants of health. Second, the propensity score-matched sensitivity analysis substantially reduced the analytical sample size and statistical power, precluding additional adjustment for residual differences. Some residual imbalance remained after matching, particularly for LEDD and height, and residual confounding by these factors therefore cannot be excluded when interpreting the matched analysis. As the matched sample represents participants within the region of common support rather than the full cohorts, these findings should be interpreted as a sensitivity analysis. Nevertheless, the similarity of the estimated between-cohort differences observed in the primary and matched analyses supports the robustness of the main findings. Additionally, medication-state differences during MDS-UPDRS III assessment may limit the comparability of clinical severity scores used for adjustment. Furthermore, freezing of gait (FOG) was not systematically assessed in either cohort. As FOG can profoundly affect walking characteristics (Mancini et al., 2019), its absence represents an important unmeasured confounder that could account for some observed DMO differences. However, the DMO differences reported here should not be interpreted as indirect evidence of differing FOG prevalence, as these metrics are not specific to freezing episodes. Future studies should include standardized FOG assessment to better understand its contribution to real-world mobility differences.

## 5 Conclusion

Overall, DMOs were able to detect differences in real-world walking behavior between PD cohorts from different settings, suggesting their potential as a scalable tool for clinical monitoring and global health research, provided that foundational efforts to improve awareness, diagnosis, and healthcare access are advanced in parallel. Digital data collection in this study showed strong compliance, good wear-time, and acceptable levels of usability and acceptability, although comfort and privacy concerns remain to be addressed. The substantially lower mobility observed in the participants with PD from Ghana, not fully explained by clinical or demographic variables, may indicate that context-sensitive approaches to diagnosis, care and intervention are needed for diverse global populations.

## Data Availability

The raw data supporting the findings of this study are available upon reasonable request, subject to ethical and privacy considerations.
