# Supplementary material for: Digital mobility assessment using wearable devices in people with Parkinson’s disease: a feasibility and implementation study in Ghana (TraPCAf) compared with a UK cohort
Source: Front Aging Neurosci. 2026 Oct 2;18:1899045. doi: 10.3389/fnagi.2026.1899045 (PMC13672635; doi:10.3389/fnagi.2026.1899045)
Supplement: Supplementary file 1 [file Data_Sheet_1.docx]

**Figure S1**. Boxplots and distributions of volume and pattern of walking activity outcomes. GHA-PD and UK-PD refer to the people with Parkinson’s disease from Ghana and United Kingdom, respectively, while GHA-CL and UK-CL refer to the healthy controls from the same countries. Outcomes with significant differences found by one-way ANOVA or Kruskall Wallis tests (p<0.05) were denoted by §. Group differences found by post hoc tests (p<0.05) were shown with connecting lines between groups’ boxplots, with corresponding p-values and effect sizes (Cohen’s d) shown above each comparison.

**Figure S2**. Boxplots and distributions of pace, rhythm and variability. GHA-PD and UK-PD refer to the people with Parkinson’s disease from Ghana and United Kingdom, respectively, while GHA-CL and UK-CL refer to the healthy controls from the same countries. Outcomes with significant differences found by one-way ANOVA or Kruskall Wallis tests were denoted by §. Group differences found by post hoc tests were shown with connecting lines between groups’ boxplots, with corresponding p-values and effect sizes (Cohen’s d) shown above each comparison.

**Figure S3**. Radar plots showing walking outcome comparisons. GHA-PD and UK-PD refer to the people with Parkinson’s disease from Ghana and United Kingdom, respectively, while GHA-CL and UK-CL refer to the healthy controls from the same countries. The plots are shown for the relevant cohorts (GHA-CL vs GHA-PD, CVS-PD vs GHA-PD, GHA-CL vs CVS-CL and CVS-CL vs CVS-PD). The central dotted line in each subplot represents corresponding reference data. Radial distance from the center represents the number of standard deviations by which the comparison group differs from the reference group. Bold text denotes outcomes that reached statistical significance within the comparison groups.

**Figure S4**. Radar plot showing pace domain outcome comparisons. GHA-PD and UK-PD refer to the people with Parkinson’s disease from Ghana and United Kingdom, respectively, while GHA-CL and UK-CL refer to the healthy controls from the same countries. The plots are shown for the relevant cohorts (GHA-CL vs GHA-PD, CVS-PD vs GHA-PD, GHA-CL vs CVS-CL and CVS-CL vs CVS-PD). The central dotted line in each subplot represents corresponding reference data. Radial distance from the center represents the number of standard deviations by which the comparison group differs from the reference group. Bold text denotes outcomes that reached statistical significance within the comparison groups.

**Figure S5**. Radar plot showing rhythm and variability domain outcome comparisons. GHA-PD and UK-PD refer to the people with Parkinson’s disease from Ghana and United Kingdom, respectively, while GHA-CL and UK-CL refer to the healthy controls from the same countries. The plots are shown for the relevant cohorts (GHA-CL vs GHA-PD, CVS-PD vs GHA-PD, GHA-CL vs CVS-CL and CVS-CL vs CVS-PD). The central dotted line in each subplot represents corresponding reference data. Radial distance from the center represents the number of standard deviations by which the comparison group differs from the reference group. Bold text denotes outcomes that reached statistical significance within the comparison groups.

**Table S1**. Covariate balance under alternative matching strategies explored for the GHA-PD and UK-PD cohorts. Values are absolute standardised mean differences after matching. Lower values indicate better balance. Propensity scores were estimated using logistic regression, followed by 1:1 nearest-neighbour matching without replacement using a caliper of 0.2 standard deviations.

| Matching Strategy | Matched Pairs (n) | Age | Gender | Height | LEDD | MDS-UPDRS Part II | MDS-UPDRS Part III | MoCA |
| --- | --- | --- | --- | --- | --- | --- | --- | --- |
| Original age-gender matched cohort | 50 | 0.067 | 0.000 | 0.606 | 0.493 | 0.911 | 0.897 | 0.840 |
| Age + MDS-UPDRS Part II | 27 | 0.010 | 0.037 | 0.561 | 1.698 | 0.050 | 0.425 | 0.799 |
| Age + MDS-UPDRS Parts II and III | 23 | 0.016 | 0.000 | 0.491 | 1.566 | 0.114 | 0.160 | 0.705 |
| Age + MDS-UPDRS Parts II and III + MoCA | 20 | 0035 | 0.050 | 0.653 | 2.122 | 0.031 | 0.020 | 0.117 |

**Table S2**. Sensitivity analyses of cohort differences in digital mobility outcomes (DMOs) using the propensity score-matched cohort. Values represent mean within-pair differences (Ghana – UK) obtained from paired Student's t-tests. Negative values indicate lower DMOs in the Ghana cohort. Two-sided 95% confidence intervals and Holm-adjusted p-values are reported.

|  | **Mean paired difference (Ghana – UK )** | **95% CI** | **Holm adjusted p-value** |
| --- | --- | --- | --- |
| **Volume** |  |  |  |
| Number of steps (steps/day) | -3459 | **-5346,-1571** | 0.019 |
| Walking Duration (hour/day) | -0.50 | **-0.82,-0.19** | 0.046 |
| **Pattern** |  |  |  |
| Number of WBs (WBs/day) | -167 | **-242,-91** | 0.004 |
| Number of WBs >10s (WBs/day) | -51 | **-83,-18** | 0.051 |
| Number of WBs >30s (WBs/day) | -2 | -8,5 | 1.000 |
| Number of WBs >60s (WBs/day) | 1 | -1,4 | 0.724 |
| Mean Daily WB Duration (sec) | 1.06 | **0.29,1.83** | 0.096 |
| Maximal Daily WB Duration (sec) | 16.49 | **2.75,30.23** | 0.169 |
| WB Duration Variability (%) | -11 | -73,50 | 1.000 |
| **Pace** |  |  |  |
| Walking Speed in WBs between 10s-30s (m/s) | -0.16 | **-0.24,-0.08** | 0.008 |
| Walking Speed in WBs >30s (m/s) | -0.26 | **-0.38,-0.13** | 0.008 |
| Maximal Walking Speed in WBs >10s (m/s) | -0.18 | **-0.32,-0.03** | 0.160 |
| Maximal Walking Speed in WBs >30s (m/s) | -0.25 | **-0.40,-0.09** | 0.051 |
| Stride Length in WBs between 10s-30s (m) | -0.16 | **-0.24,-0.08** | 0.009 |
| Stride Length in WBs >30s (m) | -0.23 | **-0.36,-0.10** | 0.024 |
| **Rhythm** |  |  |  |
| Cadence in all WBs (steps/m) | -4 | **-8,-1** | 0.169 |
| Cadence in WBs >30s (steps/m) | -8 | **-12,-3** | 0.039 |
| Maximal Cadence in WBs >30s (steps/m) | -3 | -8,1 | 0.598 |
| Stride Duration in all WBs (sec) | 0.10 | **0.06,0.15** | 0.002 |
| Stride Duration in WBs >30s (sec) | 0.15 | **0.09,0.22** | 0.001 |
| **Variability** |  |  |  |
| Walking Speed between WBs >30s (%) | 3 | -0,6 | 0.323 |
| Stride Length between WBs >30s (%) | 3 | **0,5** | 0.230 |
| Cadence between all WBs (%) | 2 | **1,3** | 0.096 |
| Stride Duration between all WBs (%) | 3 | **2,4** | 0.001 |
